# Development and Internal Validation of a Model for Predicting Internet Gaming Disorder Risk in Adolescents and Children

**DOI:** 10.3389/fpsyt.2022.873033

**Published:** 2022-06-09

**Authors:** Jiangyue Hong, Jinghan Wang, Wei Qu, Haitao Chen, Jiaqi Song, Meng Zhang, Yanli Zhao, Shuping Tan

**Affiliations:** Peking University HuiLongGuan Clinical Medical School, Beijing HuiLongGuan Hospital, Beijing, China

**Keywords:** Internet gaming disorder, children, prediction model, adolescents, nomogram

## Abstract

**Background:**

The high prevalence of Internet gaming disorder among children and adolescents and its severe psychological, health, and social consequences have become a public emergency. A high efficiency and cost-effective early recognition method are urgently needed.

**Objective:**

We aim to develop and internally validate a nomogram model for predicting Internet gaming disorder (IGD) risk in Chinese adolescents and children.

**Methods:**

Through an online survey, 780 children and adolescents aged 7–18 years who participated in the survey from June to August 2021 were selected. The least absolute shrinkage and selection operator regression model was used to filter the factors. Multivariate logistic regression analysis was used to establish the prediction model and generate nomograms and a website calculator. The area under the receiver operating characteristic curve, calibration plot, and decision curve analysis were used to evaluate the model's discrimination, calibration, and clinical utility. Bootstrapping validation was used to verify the model internally.

**Results:**

Male sex and experience of game consumption were the two most important predictors. Both models exhibited good discrimination, with an area under the curve >0.80. The calibration plots were both close to the diagonal line (45°). Decision curve analyses revealed that two nomograms were clinically useful when the threshold probability for the intervention was set to 5–75%.

**Conclusion:**

Two prediction models appear to be reliable tools for Internet gaming disorder screening in children and adolescents, which can also help clinicians to personalize treatment plans. Moreover, from the standpoint of simplification and cost, Model 2 appears to be a better alternative.

## Introduction

Internet gaming disorder (IGD) was listed as a clinical phenomenon requiring further research by the Diagnostic Statistical Manual of Mental Disorders (Fifth Edition, DSM-5) in 2013 (1). Compared with adults, children and adolescents have a higher prevalence of IGD, by more than 20%, in many regions (2, 3). At present, the diagnosis of IGD is usually determined by doctors There have been many studies on the predictors of IGD. For example, Rho (4) found that greater amounts of time gaming, video game consumption experience, and single marital status were important predictors of IGD. Estévez (5) pointed out that difficulty in emotion regulation and lower peer attachment have predictive effects on problematic Internet gaming. In a survey conducted in a Korean population, Young Choi found that high impulsivity, high game cost, and long gaming time during workdays were significant risk factors for IGD (6). Jeong et al. (7) found that longer average daily gaming time, playing multiplayer games, depressive symptoms, and hyperactivity symptoms were independent risk factors for IGD. A longitudinal study conducted by Wartberg et al. (8) found that male sex, severe self-esteem problems, and severe hyperactivity symptoms are predictors of problematic online gaming behavior. In addition to the above, several other studies focusing on risk factors for IGD support that many variables are significantly associated with the development of IGD, including video game cost (9, 10), gaming time (11, 12), difficulty in emotion regulation (13), higher impulsivity (11, 14, 15), hyperactivity symptoms (16–19), self-esteem problems (19–21), playing multiplayer games (22, 23), emotional symptoms (24, 25), peer relationship problems (26–28), family relationship problems (29, 30), and male sex (2, 12, 31–33), among others. Therefore, we collected information on these variables, with age and lifestyle factors (which we believe may be related to family support) as candidate predictors.

To our knowledge, no prediction model has been developed to assess the risk of IGD in children and adolescents. Therefore, this study established a prediction model and conducted an internal validation to identify individuals at high risk of IGD, which is helpful in implementing the targeted intervention promptly.

## Materials and Methods

### Participants

From June 2021 to August 2021, we recruited participants through convenient sampling and online survey. The inclusion criteria were as follows: (1) Chinese adolescents who voluntarily participated in the study; (2) Age 7–18 years old; (3) Could use mobile phones independently (IOS or Android system); (4) Participants and guardians were required to submit informed consent online. The exclusion criteria were: (1) not completing all questionnaires; (2) exceeding the response duration (mean ± three standard deviations). This study was approved by the ethics committee of Beijing Huilongguan Hospital. All subjects were informed of the study plan before enrollment and submitted informed consent online.

### Measures

#### Self-Administered Video Game Habit Questionnaire

We used a self-administered questionnaire on gaming habits to identify predictors related to video gaming among participants. The questionnaire included seven items: the age at the start of contact with video games, the age at the start of habitually playing video games, the average daily video game time in the preceding 6 months, the number of frequently played games (1–2 / 3 and above), the main ways of playing games (team games/single-player games), whether there was an experience of video game consumption (no/yes), and percentage of monthly game consumption in total expenditure (<20% / ≥20%).

#### IGD Scale

The outcome variable in the model is IGD, which is self-rated by the IGD scale. The scale was compiled by Pontes et al. (34), according to the diagnostic criteria of IGD proposed in the DSM-V. After being translated and appropriately modified by Chinese scholar Hong et al. (35) a Chinese version of the IGD Scale was developed The scale has good reliability and validity, with a Cronbach's α coefficient of 0.90 and a split-half reliability coefficient of 0.88 (35). There are nine items rated on a 5-point Likert-type scale ranging from 1 (never) to 5 (always). Those with a total score >21 (36) were defined as having IGD.

#### Strength and Difficulties Questionnaire

The questionnaire was compiled by American psychologist Robert Goodman in 1997 (37), and later translated into Chinese by the Shanghai Mental Health Center (38). The subscales of emotional problems, hyperactivity, and peer problems were used to evaluate the subjects. Each subscale had five items, with a score of 0–2 (non-conforming, slightly conforming, and fully conforming), ranging from 0 to 10. Higher scores indicated more severe difficulties. The Cronbach's α coefficient of the questionnaire was 0.79 (38).

#### The Self-Esteem Scale

The Self-Esteem Scale was originally created by Rosenberg (39). After being translated and revised by Ji et al. (40), a Chinese version was developed. This scale was used to evaluate the degree of self-esteem. The scale has 10 items, which are rated from 1 to 4 (very inconsistent, inconsistent, consistent, and very consistent) for a total score ranging from 10–40. A higher score indicates a higher degree of self-esteem. The split-half reliability coefficient of the scale was 0.96, the test-retest reliability coefficient was 0.78, and the criterion validity coefficient was 0.52 (41).

#### The Difficulties in Emotion Regulation Scale

DERS was compiled by Gratz and Roemer (42) and later translated into Chinese by Wang et al. (43). The scale includes six dimensions: lack of emotional clarity (clarity, five items), impulse control difficulties (impulse, six items), difficulties in goal-directed behavior when emotionally present (goals, five items), difficulty in accepting emotional responses (non-acceptance, six items), limited access to emotion regulation strategies (strategies, eight items), and lack of emotional awareness (awareness, five items). Items are rated on a 5-point Likert scale ranging from 1 (almost never) to 5 (almost always). Higher scores indicate more severe difficulties of emotion regulation. Cronbach's α coefficient of the scale was between 0.88 and 0.96, and the test-retest reliability was between 0.52 and 0.77 (44).

#### Brief Sensation Seeking Scale–Chinese Version

The BSSS was developed by Hoyle (45) and later translated into Chinese by Chen et al. (46). The scale is used to evaluate an individual's sensory seeking traits, including four dimensions: experience seeking, boredom susceptibility, thrill and adventure seeking, and disinhibition. Each dimension has two items, which are rated using a 5-point scale (1 = completely inconsistent, 5 = completely consistent) for a total score of 2–10. Higher scores indicate more pronounced traits. The Cronbach's α coefficient was 0.82, and the test-retest reliability coefficient was 0.69 (46).

#### Family Environment Scale-Chinese Version

In 1981, the Family Environment Scale was compiled by Moss and Moss (47). Fei et al. (48) translated it into Chinese in 1991. The subscales of cohesion, expressiveness, and conflict were selected to evaluate the participants. Each subscale had nine true-false questions. The subscale scores ranged from 0–9, with higher scores indicating more noticeable family's environmental characteristics. The reliability of each scale dimension was approximately 0.6 (49).

### Statistical Analysis

Statistical analysis was performed using Empowerstats software and R software 4.1.1. Relevant software packages include rms, rmda, pROC, DynNom, and pmsampsize. Continuous variables are expressed as median (range) and were compared using the Mann–Whitney U test. In addition, the χ2 test was used to calculate statistical differences in categorical variables.

**Figure 1:**
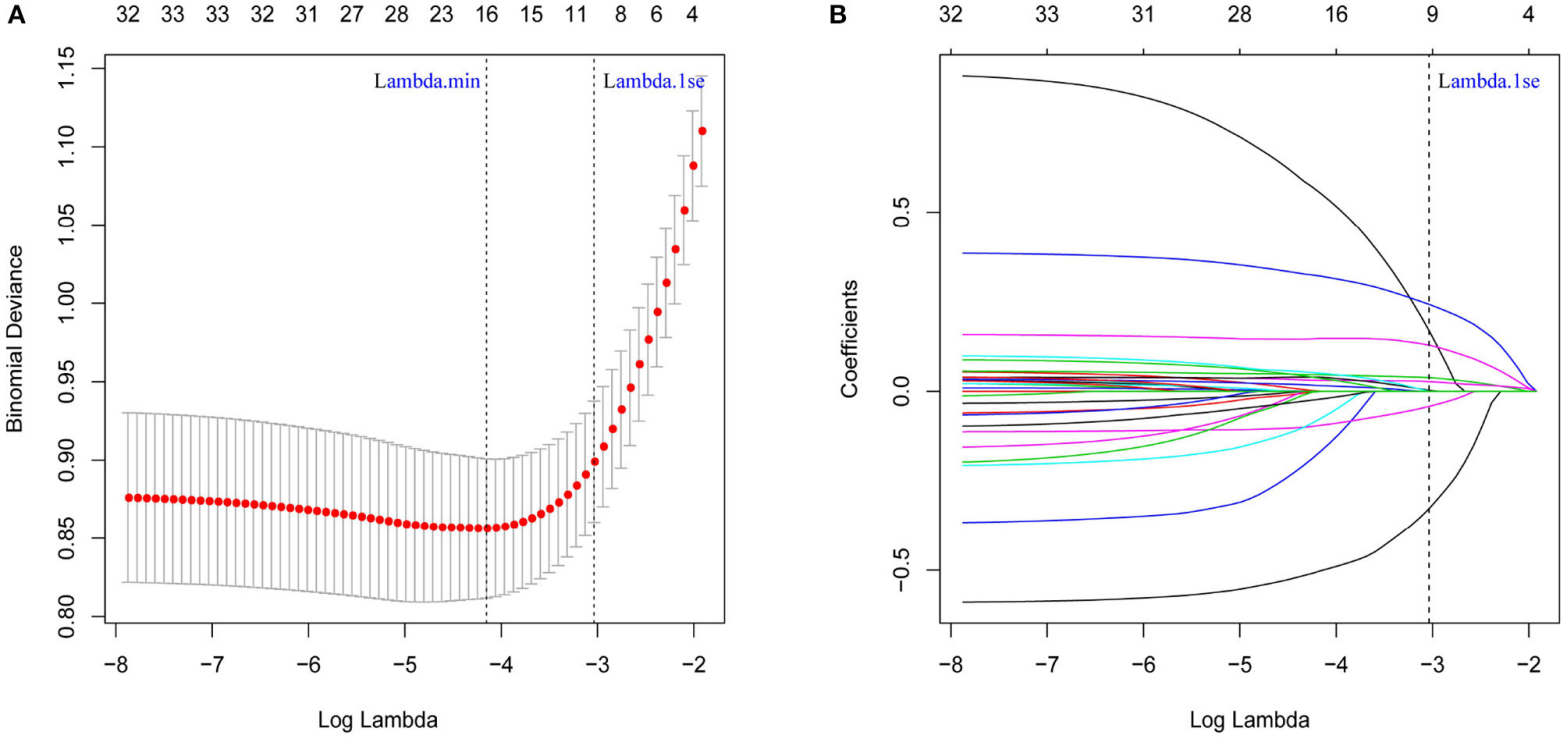
Determination of Internet Gaming disorder risk factors in children and adolescents by least absolute shrinkage and selection operator (LASSO) regression analysis. **(A)** The cross-validation for LASSO regression, where the parameter lambda was adjusted to find the best function set, is shown. The vertical dotted line on the left panel represents the log (lambda) corresponding to the optimal lambda. **(B)** The coefficients of predictors changed with lambda. The vertical dotted line in the right panel corresponds to the eight features selected with non-zero LASSO cross-validation coefficients.

The least absolute shrinkage and selection operator (LASSO) regression was used to screen the predictors. The idea is to construct a penalty function to compress the regression coefficients of variables and shrink some variable coefficients to zero to achieve the screening of variables. Lambda is the penalty coefficient filtered through a 10-fold cross-validation (Figure 1A). Lambda.min refers to the penalty coefficient that minimizes the average error of the model. Log (lambda.min) = −4.15, draws the vertical line, and the number of variables screened was 14. Lambda.1se refers to the maximum penalty coefficient that makes the average error of the model within one standard deviation of the minimum value. Log (lambda.1se) = −3.04, draw a vertical line, and the number of variables screened is 8. To make the model error within the acceptable range and thus make the model concise and convenient for clinical use, lambda.1se was selected as the optimal penalty coefficient (Figure 1B).

The predictors screened by LASSO regression were included in the multivariate logistic regression analysis. Model 1 (complete) and Model 2 (simplified) were established. A logistic regression model was used to create a nomogram to predict the risk of IGD. The model's performance was internally validated using the bootstrap self-sampling method (k = 1,000). The area under the receiver operating characteristic curve (AUC) was used to evaluate the discrimination of the model. The closer the AUC is to 1, the better the model's discrimination. The Delong method was used to compare the difference between the two models. A calibration plot was used to evaluate the calibration of the model (50). Ideally, the calibration plot was a straight line with a slope of 1 and an intercept of 0. The more consistent the calibration plot of the model is with the reference line, the higher the calibration degree of the model. Decision curve analysis (51) was used to evaluate the clinical usefulness of the nomogram by calculating the net benefit under different threshold probabilities. The net benefit equals the true-positive rate multiplied by the gain of receiving treatment minus the false-positive rate multiplied by the loss of receiving treatment. Statistical significance was set at *P* < 0.05.

## Results

### Participant Characteristics

According to the IGD Scale scores, participants were divided into the IGD (*n* = 190, 24.3%) and non IGD (*n* = 590, 75.5%) groups. The IGD group included 124 boys and 66 girls, aged 7–18 years, with an average age of 14.4 years and an average length of education of 8.3 years. The non-IGD group included 265 boys and 325 girls, aged 7 to 18 years, with an average age of 14.3 years and an average length of education of 8.3 years. Table 1 lists the detailed demographics, video game use, and psychological characteristics.

**Table 1:** Comparison of clinical characteristics between participants with and without IGD [*n* (%), median (range)].

	**Total** **(*n* = 780)**	**non-IGD** **(*n* = 590)**	**IGD** **(*n* = 190)**	***P-*value**
Age (years)	14.0 (7.0, 18.0)	14.0 (7.0, 18.0)	15.0 (7.0, 18.0)	0.407
Sex				<0.001
Male	389 (49.9%)	265 (44.9%)	124 (65.3%)	
Female	391 (50.1%)	325 (55.1%)	66 (34.7%)	
Education (years)	8.0 (1.0, 13.0)	8.0 (1.0, 13.0)	8.0 (1.0, 12.0)	0.492
Lifestyle factors				<0.001
Boarding school	710 (91.0%)	550 (93.2%)	160 (84.2%)	
Non-Boarding school	70 (9.0%)	40 (6.8%)	30 (15.8%)	
Age at the start of contact with video games (years)	11.0 (3.0, 18.0)	11.0 (3.0, 18.0)	10.0 (3.0, 16.0)	<0.001
Age at the start of habitually playing video games games (years)	12.0 (3.0, 18.0)	12.0 (3.0, 18.0)	11.0 (3.0, 17.0)	<0.001
Average daily video game time (hours)	0.9 (0.0, 7.0)	0.6 (0.0, 7.0)	1.3 (0.0, 7.0)	<0.001
Number of frequently played games				<0.001
1 or 2	629 (80.6%)	503 (85.3%)	126 (66.3%)	
3 and above	151 (19.4%)	87 (14.7%)	64 (33.7%)	
Main ways of playing games				<0.001
Single-player games	434 (55.6%)	351 (59.5%)	83 (43.7%)	
Team games	346 (44.4%)	239 (40.5%)	107 (56.3%)	
Previous experience of game consumption				<0.001
No	476 (61.0%)	404 (68.5%)	72 (37.9%)	
Yes	304 (39.0%)	186 (31.5%)	118 (62.1%)	
Percentage of monthly video game consumption in total expenditure				<0.001
<20%	719 (92.2%)	561 (95.1%)	158 (83.2%)	
≥20%	61 (7.8%)	29 (4.9%)	32 (16.8%)	
Emotional problems	3.0 (0.0, 10.0)	2.0 (0.0, 10.0)	4.0 (0.0, 10.0)	<0.001
Hyperactivity	4.0 (0.0, 10.0)	3.0 (0.0, 10.0)	5.0 (0.0, 10.0)	<0.001
Peer problems	3.0 (0.0, 9.0)	3.0 (0.0, 9.0)	4.0 (0.0, 8.0)	<0.001
Self-esteem	28.0 (12.0, 40.0)	29.0 (12.0, 40.0)	27.0 (16.0, 38.0)	<0.001
Lack of emotional awareness	18.0 (6.0, 30.0)	18.0 (6.0, 30.0)	19.0 (6.0, 30.0)	0.074
Impulse control difficulties	11.0 (6.0, 30.0)	10.0 (6.0, 29.0)	14.0 (6.0, 30.0)	<0.001
Difficulties engaging in goal-directed behavior when emotionally aroused	13.0 (5.0, 25.0)	12.0 (5.0, 25.0)	15.0 (6.0, 25.0)	<0.001
Difficulty accepting emotional responses	12.0 (6.0, 30.0)	11.0 (6.0, 30.0)	13.0 (6.0, 30.0)	<0.001
Limited access to emotion regulation strategies	16.0 (8.0, 40.0)	15.0 (8.0, 39.0)	20.0 (8.0, 40.0)	<0.001
Lack of emotional clarity	12.0 (5.0, 25.0)	11.0 (5.0, 23.0)	13.0 (5.0, 25.0)	<0.001
Experience seeking	6.0 (2.0, 10.0)	6.0 (2.0, 10.0)	6.0 (2.0, 10.0)	0.329
Boredom susceptibility	6.0 (2.0, 10.0)	5.0 (2.0, 10.0)	6.0 (2.0, 10.0)	<0.001
Thrill and adventure seeking	6.0 (2.0, 10.0)	6.0 (2.0, 10.0)	6.0 (2.0, 10.0)	0.053
Disinhibition	3.0 (2.0, 10.0)	3.0 (2.0, 10.0)	4.0 (2.0, 10.0)	<0.001
Family cohesion	7.0 (0.0, 9.0)	7.0 (0.0, 9.0)	6.0 (0.0, 9.0)	<0.001
Family expressiveness	5.0 (0.0, 9.0)	5.0 (0.0, 8.0)	5.0 (1.0, 9.0)	<0.001
Family conflict	3.0 (0.0, 8.0)	3.0 (0.0, 8.0)	4.0 (0.0, 8.0)	<0.001

*P: categorical variables—χ2 test; continuous variables—Mann–Whitney U test*.

### Development of Nomograms for IGD Risk Prediction

Based on lasso regression analysis, the following eight predictors were selected: male sex, average daily video game time, experience of video game consumption, hyperactivity, age at the start of contact with video games (years), impulse control difficulties, limited access to emotion regulation strategies, and disinhibition.

Logistic regression was used to establish prediction models. Model 1 includes the above eight predictors, as presented in Table 2. Among them, male sex (OR = 2.17; 95%CI 1.42–3.32) and having video game consumption experience (OR = 1.93; 95% CI1.29–2.90) were the two most important predictors. To further simplify the model and keep the AUC value above 0.8, after constructing the model using different combinations of predictors, Model 2 was selected. Model 2 includes only four predictors: male sex, average daily video game time, having video game consumption experience, and hyperactivity, as presented in Table 2. Male sex (OR = 2.06; 95%CI 1.39–3.04) and having video game consumption experience (OR = 2.10; 95%CI 1.42–3.08) remained the two most important predictors.

**Table 2:** Risk factors for IGD identified by multivariable logistic analysis.

**Factors**	**Model 1**			**Model 2**		
	**OR**	**95%CI**	***P-*value**	**OR**	**95%CI**	***P-*value**
Male sex	2.17	(1.42–3.32)	<0.001	2.06	(1.39–3.04)	<0.001
Previous experience of video game consumption	1.93	(1.29–2.90)	<0.001	2.10	(1.42–3.08)	<0.001
Average daily video game time (hours)	1.47	(1.26–1.73)	0.001	1.53	(1.32–1.79)	<0.001
Hyperactivity	1.25	(1.11–1.41)	<0.001	1.49	(1.35–1.65)	<0.001
Age at the start of contact with video games (years)	0.88	(0.82–0.95)	<0.001			
Limited access to emotion regulation strategies	1.05	(1.00–1.10)	0.027			
Impulse control difficulties	1.07	(1.00–1.13)	0.027			
Disinhibition	1.10	(0.97–1.23)	0.141			

*OR, odds ratio; CI, confidence interval*.

**Figure 2:**
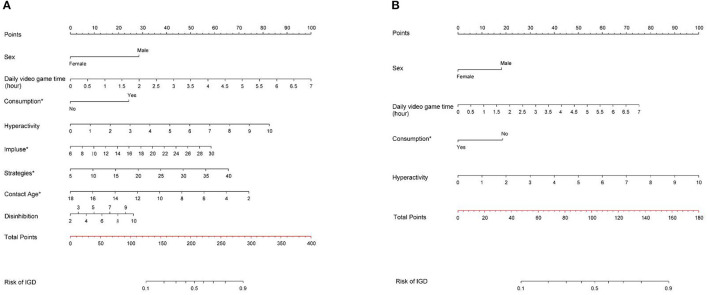
Nomograms for predicting the risk of Internet gaming disorder in children and adolescents. **(A)** Model 1; **(B)** Model 2. Consumption*: Experience of video game consumption; Impulse*: Impulse control difficulties; Strategies*: limited access to emotion regulation strategies; Contact age*: Age at the start of contact with video games.

**Figure 3:**
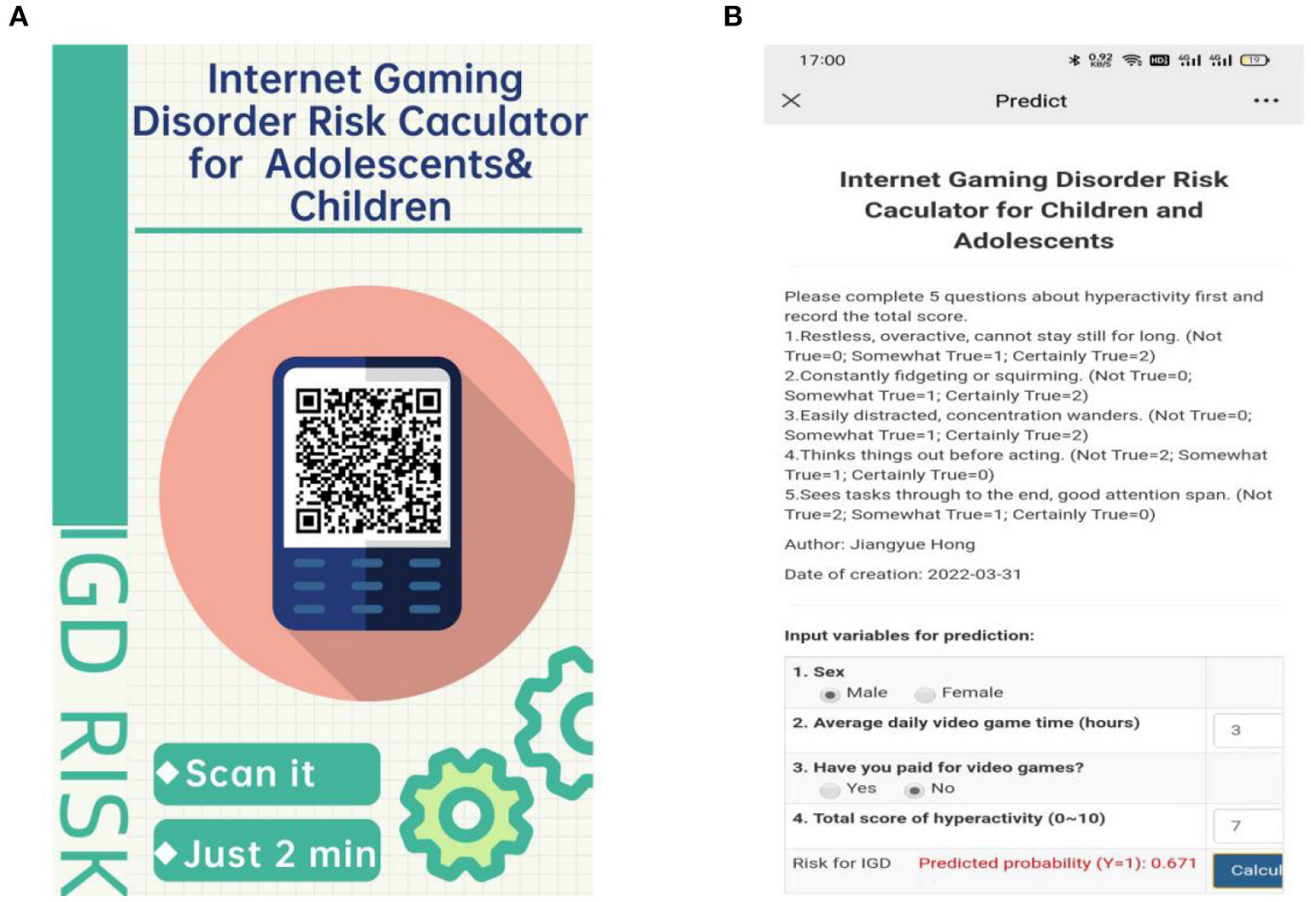
Internet gaming disorder risk calculator for adolescents and children. **(A)** QR (quick response) code poster of website calculator. **(B)** Interactive user interface.

Nomograms were established for Models 1 and 2 to calculate IGD risk, as presented in Figure 2. At the same time, based on model 2, an IGD risk website calculator was developed, as shown in Figure 3. Scan the QR code in Figure 3A or visit the website to gain free access to the calculator: http://www.empowerstats.net/pmodel/?m=22029_IGDpredictionmodel. The interactive user interface is shown in Figure 3B.

### Validation of Nomograms for IGD Risk Prediction

**Figure 4:**
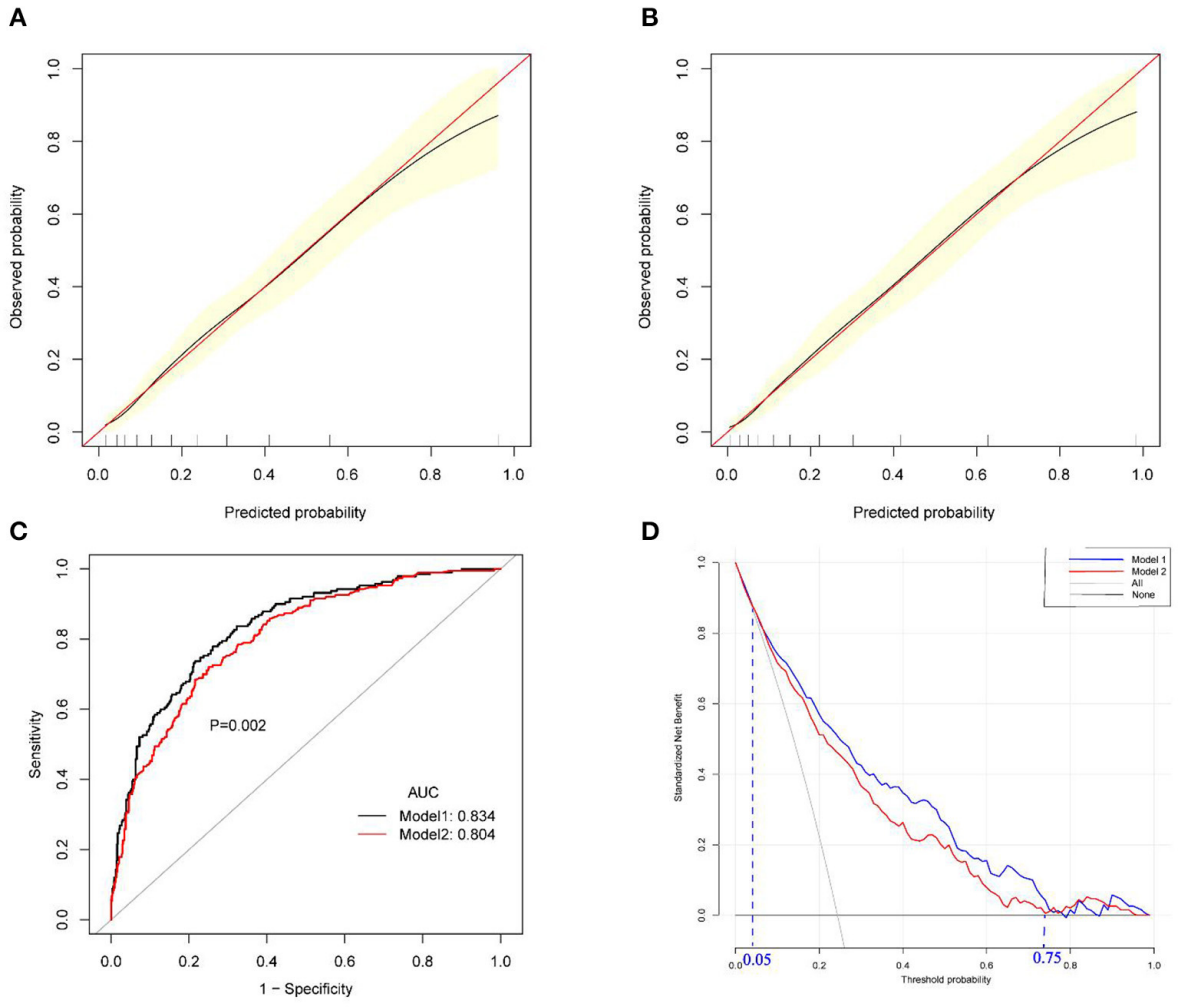
Validation and decision curve analysis of Model 1 and Model 2. **(A)** Calibration curve of Model 1; **(B)** calibration curve of Model 2; **(C)** Receiver operating characteristics curves of Model 1 and Model 2; **(D)** Decision curve analysis of Model 1 and Model 2. AUC: area under the curve.

The internal bootstrap validation demonstrated that the Model 1-derived curve fits well with the ideal curve with a probability of 0 and 0.70. However, when the probability was set to >0.70, Model 1 could overestimate the probability of IGD (Figure 4A). Model 2 had a similar tendency; however, the start point of overestimation at the predicted probability was slightly lower than 0.70 (Figure 4B). Both models 1 and 2 showed good fitting and calibration.

Furthermore, Models 1 and 2 were further validated internally using receiver operating characteristic curve. The AUC for Model 1 was 0.834 (95% CI 0.801–0.867), yielding a sensitivity of 73.7% and a specificity of 78.5% at the optimal cutoff value that maximized the Youden's index. At the optimal corresponding cutoff values, Model 2 yielded a sensitivity of 72.1% and a specificity of 74.9%, and the AUC was 0.804 (95% CI: 0.770–0.839). The two nomograms have a good ability to discriminate whether an individual suffers from IGD (Figure 4C), although the discrimination of Model 1 was better than that of Model 2 (*P* = 0.002).

### Clinical Use of Nomograms for IGD Risk Prediction

The decision curve analysis comparing the clinical usefulness of Models 1 and 2 is presented in Figure 4D. The threshold probability for IGD is plotted on the x-axis, and the model's standard net benefit is plotted on the y-axis. The area among the treat all lines, treat none line, and the model curve represent the clinical usefulness of each model. Both models 1 and 2 showed better cost-effectiveness than treating none and treating all. A comparison of the two nomogram models with two extreme schemes (“treat none” and “treat all ”) revealed a net benefit of >0 with a threshold probability of between 5 and 75%. Moreover, in this range, a smaller threshold probability led to a higher net benefit. Only when the threshold probability is <5% is there no difference between using our model and the scheme that considers treating all participants (i.e., regardless of whether they have IGD or not). On the other hand, when the threshold probability is above 75%, there is no difference between using our model and not treating any participant (i.e., no intervention regardless of IGD). Because Model 1 is located at the upper right, its clinical usefulness surpasses that of Model 2.

## Discussion

In this study, the proportion of IGD in children and adolescents was 24.3%, which is similar to that reported by Chinese scholar Zheng-Chuan (52). As children and adolescents' IGD has become a shared interest, the assessment of the risk of IGD in this population has acquired clinical significance, which will encourage the early identification of high-risk children and adolescents to carry out targeted prevention or intervention. To the best of our knowledge, no nomogram has been developed to evaluate the risk of IGD. In this study, two models were developed to predict the risk of IGD in children and adolescents, and both were verified internally. Both exhibited good discrimination, calibration, and clinical usefulness.

In the final model, male sex, and experience of video game consumption were the two most important predictors, followed by daily video game time and hyperactivity, and the weight of the remaining predictors was small. For video game consumption experience, a South Korean study reported that video game consumption was a predictor of IGD in adults aged 20–40 years (4). Our result also aligns with several studies of adolescents that suggest that in-game purchases are associated with the development of behavioral addictions, such as IGD (9) and compulsive gambling (53, 54). For example, in a study of Japanese adolescents, Soichiro Ide found that teenagers who bought loot boxes were significantly more likely to have problems with online gaming. Loot boxes, also known as “loot cases” or “loot chests,” are a central feature of many video games. Players purchase loot boxes to obtain valuable in-game items that enhance their gameplay, which in turn may intensify their involvement in video gaming. In addition, this study found that boys have a higher risk of IGD than girls, which is consistent with the results of a longitudinal study in Germany (8). The reason appears to be related to the different ways of using the Internet, as males prefer games, while females prefer social media (55). Therefore, boys are more likely to suffer from IGD, which suggests the need for greater focus on boys to identify the problematic use of video games. Previous studies have indicated that daily video game time plays an important role in predicting the occurrence of IGD (4, 6, 7). A longer daily playtime was found to increase the likelihood for an individual to suffer from IGD, which is in line with clinical findings. This suggests that strict restrictions on children's and adolescents' game time are required to reduce the possibility of IGD and reduce the time spent playing video games in favor of other beneficial activities, such as physical exercise, parent-child interaction, and sleep. Hyperactivity is closely associated with IGDs. A cross-lagged panel design study among German adolescents found that higher levels of hyperactivity could predict the occurrence of IGD 1 year later. Moreover, hyperactivity was found to be an independent risk factor for the occurrence of IGD and a predictor of the persistence of IGD. IGD patients with higher hyperactivity levels are more likely to continue suffering from IGD 2 years later (7). Therefore, from a clinical perspective, the prevention and relief of IGD may require appropriate hyperactivity treatment.

Among other predictors that could be targeted for clinical intervention, the first is the age of exposure to video games. This study suggests that an earlier exposure to video games leads to a greater likelihood of developing IGD. This may be due to the long developmental experience and late maturity of the prefrontal cortex and to the fact that the executive control ability of children and adolescents is still not mature (56). Therefore, early exposure to video games may make it difficult for children to control their gaming behavior. The executive function is not mature until late adolescence (57, 58); therefore, we suggest that parents should control their children's exposure to video games after puberty. Furthermore, impulse control difficulties and limited access to emotion regulation strategies predict IGD. Some studies have shown that people with difficulty in emotion regulation engage in addictive behaviors to escape or regulate negative feelings (59). It is also plausible that if individuals exhibit poor control over impulsive reactions when feeling negative emotions or lack emotion regulation strategies, they might more likely get involved in behaviors that prolong their positive emotional state, such as playing video games. Video games might be a means for them to elevate their mood, which increases the likelihood of indulging in virtual video games. From the perspective of interventions, in addition to providing children and adolescents with more resources and tools to help them better regulate their emotions, parents and teachers also need to pay timely attention and provide appropriate guidance.

Overall, the performances of the two models were found to be similar, but Model 1 was more complex than Model 2. Impulse control difficulties, limited access to emotion regulation strategies, and disinhibition require an evaluation with the corresponding scale and scores for incorporation into the model. This may increase the utility cost of Model 1 and reduce its convenience as a screening tool. Model 2 contained only four predictors. Although the hyperactivity score also needs to be evaluated by the scale, only five items significantly reduce the utility cost. Considering that the two models have a similar overall performance, Model 2 may be more cost effective.

This study had some limitations. First, there is a lack of heterogeneous data to externally validate the models. Further studies should externally validate the models and evaluate their stability (60). Second, the identification of IGD in this study was based on the self-rated IGD Scale, which may be less accurate than face-to-face interviews. If possible, future research can use the results of these interviews as a diagnostic criterion of IGD in a large population. Moreover, subsequent researchers should consider including more game-related variables as predictors, such as game genres (61), and conducting dynamic follow-up observations to further verify and optimize the model's clinical utility.

## Conclusion

In summary, the two nomograms developed in this study can be considered economic and effective tools for IGD risk assessment in Chinese children and adolescents. The creation of Nomograms and website calculator can objectively and accurately predict the probability of Internet gaming disorder and can help clinicians to personalize treatment plans. Moreover, from the standpoint of simplification and cost, Model 2 appears to be a better alternative.

## Data Availability Statement

The raw data supporting the conclusions of this article will be made available by the authors, without undue reservation.

## Ethics Statement

The studies involving human participants were reviewed and approved by the Ethics Committee of Beijing Huilongguan Hospital. Written informed consent to participate in this study was provided by the participants' legal guardian/next of kin.

## Author Contributions

JH prepared the first draft of the paper. JW conducted the analysis. WQ designed a mobile phone program for the online survey. HC, JS, MZ, and YZ contributed to the data cleaning and manuscript interpretation. ST supervised this research and revised the manuscript. All authors read and approved the manuscript.

## Funding

This work was funded by the Beijing Natural Science Foundation Grant (No. 7202072) and the Beijing Municipal Science & Technology Commission Grant (Z191100006619104).

## Conflict of Interest

The authors declare that the research was conducted in the absence of any commercial or financial relationships that could be construed as a potential conflict of interest.

## Publisher's Note

All claims expressed in this article are solely those of the authors and do not necessarily represent those of their affiliated organizations, or those of the publisher, the editors and the reviewers. Any product that may be evaluated in this article, or claim that may be made by its manufacturer, is not guaranteed or endorsed by the publisher.
