# Supplementary figures and images for: Development and Internal Validation of a Model for Predicting Internet Gaming Disorder Risk in Adolescents and Children
Source: Front Psychiatry. 2022 Jun 9;13:873033. doi: 10.3389/fpsyt.2022.873033 (PMC9222136; doi:10.3389/fpsyt.2022.873033)

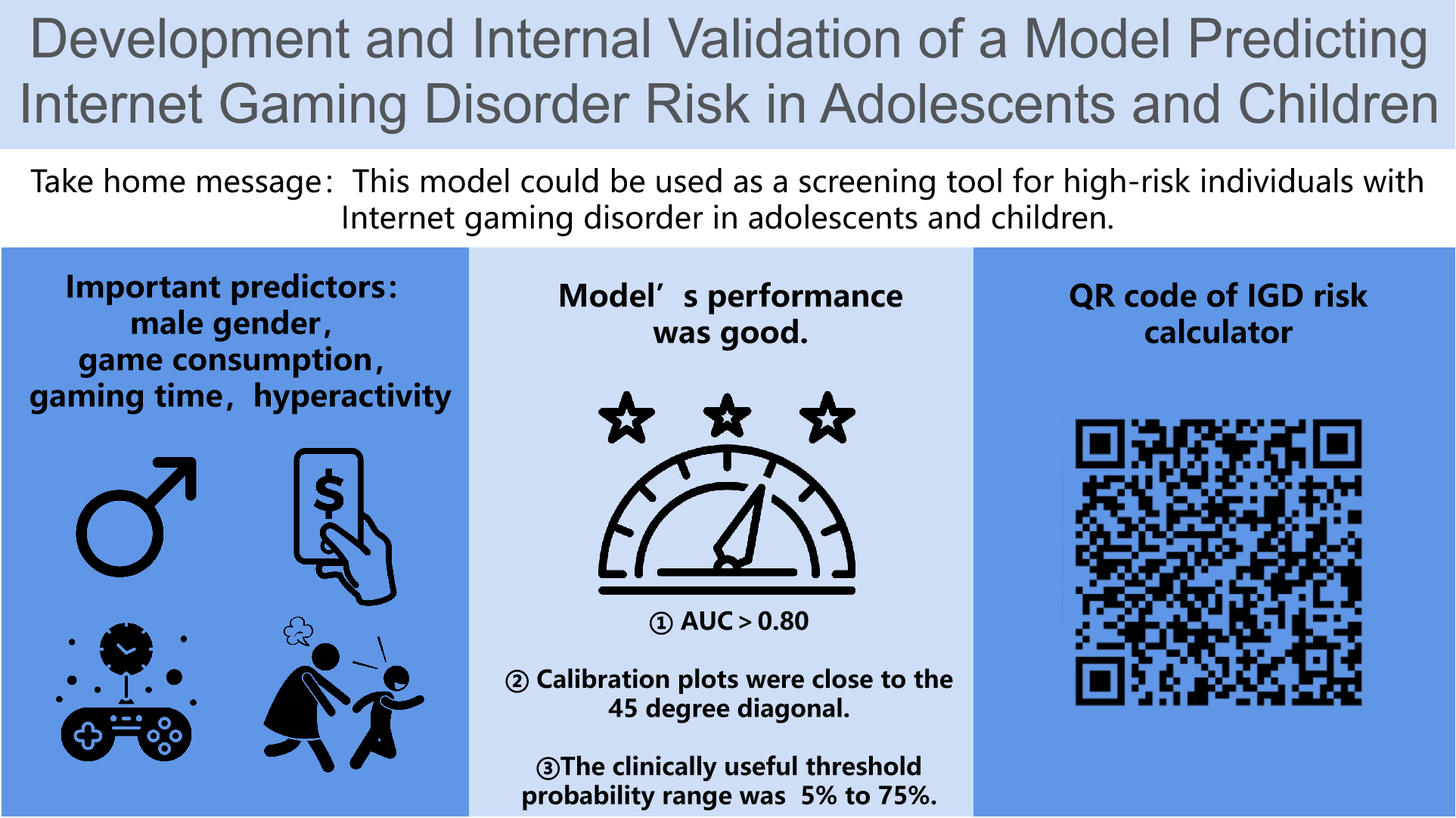

Supplement: Supplementary file 1 [file Image_1.TIF]
